# Genome-Wide Association Analysis of the Genetic Basis for Sheath Blight Resistance in Rice

**DOI:** 10.1186/s12284-019-0351-5

**Published:** 2019-12-18

**Authors:** Fan Zhang, Dan Zeng, Cong-Shun Zhang, Jia-Ling Lu, Teng-Jun Chen, Jun-Ping Xie, Yong-Li Zhou

**Affiliations:** Institute of Crop Sciences/The National Key Facility for Crop Gene Resources and Genetic Improvement, Chinese Academy of Agricultural Sciences, 12 South Zhong-Guan-Cun Street, Haidian District, Beijing, 100081 China

**Keywords:** Sheath blight resistance, Germplasm, GWAS, Rice

## Abstract

**Background:**

Sheath blight (ShB), caused by *Rhizoctonia solani* Kühn, is one of the most destructive rice diseases. Developing ShB-resistant rice cultivars represents the most economical and environmentally sound strategy for managing ShB.

**Results:**

To characterize the genetic basis for ShB resistance in rice, we conducted association studies for traits related to ShB resistance, namely culm length (CL), lesion height (LH), and relative lesion height (RLH). Combined a single locus genome-wide scan and a multi-locus method using 2,977,750 single-nucleotide polymorphisms to analyse 563 rice accessions, we detected 134, 562, and 75 suggestive associations with CL, LH, and RLH, respectively. The adjacent signals associated with RLH were merged into 27 suggestively associated loci (SALs) based on the estimated linkage disequilibrium blocks. More than 44% of detected RLH-SALs harboured multiple QTLs/genes associated with ShB resistance, while the other RLH-SALs were putative novel ShB resistance loci. A total of 261 ShB resistance putative functional genes were screened from 23 RLH-SALs according to bioinformatics and haplotype analyses. Some of the annotated genes were previously reported to encode defence-related and pathogenesis-related proteins, suggesting that quantitative resistance to ShB in rice is mediated by SA- and JA-dependent signalling pathways.

**Conclusions:**

Our findings may improve the application of germplasm resources as well as knowledge-based ShB management and the breeding of ShB-resistant rice cultivars.

## Background

Sheath blight (ShB), caused by *Rhizoctonia solani* Kühn (*R. solani*), is one of the most destructive rice diseases (Zheng et al. 2013). This disease, which is prevalent in East Asia and the southern USA (Chen et al. 2012; Prasad and Eizenga 2008), results in heavy rice yield losses when nitrogen fertilizers are extensively applied (Savary et al. 1995). *R. solani* is a soil-borne hemibiotrophic pathogen (Kouzai et al. 2018) that survives as sclerotia or mycelia in the debris of host plants during its necrotrophic phase. To date, no major ShB resistance genes or rice cultivars exhibiting complete resistance to *R. solani* have been reported, likely because of the polygenic nature of ShB resistance. The application of chemicals remains the major method for controlling rice ShB. However, the overuse of chemical fungicides contributes to increased health risks and environmental problems (Zeng et al. 2011). Therefore, developing ShB-resistant rice cultivars represents the most economical and environmentally sound strategy for managing ShB (Liu et al. 2009).

Following the detection of the first quantitative trait locus (QTL) for ShB resistance in rice (Li et al. 1995), more than 60 QTLs conferring ShB resistance have been detected among the 12 rice chromosomes based on bi-parental genetic mapping populations (Taguchi-Shiobara et al. 2013; Wen et al. 2015; Yadav et al. 2015; Zeng et al. 2011; Zeng et al. 2015). Of these, only *qSBR11–1* and *qSB-11*^LE^ have been fine-mapped (Channamallikarjuna et al. 2010; Zuo et al. 2013). Additionally, a rice chitinase gene (*LOC*_*Os11g47510*), which was cloned from the *qSBR11–1* region of an *R. solani*-tolerant rice line (Tetep), was functionally validated by the genetic transformation of an ShB-susceptible *japonica* rice line (Taipei 309) (Richa et al. 2017). Moreover, some major QTLs associated with ShB resistance, such as *qSB-9*^Tq^, have been used in breeding programmes (Taguchi-Shiobara et al. 2013; Zuo et al. 2008). Pyramiding diverse ShB resistance alleles from QTLs differing in their level of moderate resistance by marker-assisted selection can efficiently enhance the resistance of rice to *R. solani* (Hossain et al. 2016; Yadav et al. 2015). However, the resistance level of a near-isogenic line containing three ShB resistance alleles was not significantly higher than that of a line containing two ShB resistance alleles (Zeng et al. 2011).

A genome-wide association study (GWAS) of natural populations involving high-density single nucleotide polymorphisms (SNPs) detected by next-generation sequencing was used to dissect the genetic architecture of blast resistance (Kang et al. 2016) and bacterial blight resistance (Zhang et al. 2017) in rice. There were only two reports about the identification of ShB resistance QTLs by GWAS (Jia et al. 2012; Chen et al. 2019). A total of 10 ShB resistance QTLs were identified by an association mapping study involving 217 sub-core entries from the United States Department of Agriculture rice core collection and 155 simple sequence repeat markers (Jia et al. 2012). Recently, at least 11 SNP loci significantly associated with SB resistance at the seedling stage under artificial inoculation were detected by GWAS using 299 diverse rice varieties from the rice diversity panel with genotyping by 44,000 SNP markers array (Chen et al. 2019). However, no association studies have evaluated the diverse rice germplasm to identify potentially novel ShB resistance loci at the tillering stage based on genome-wide high-density SNPs. As part of the 3000 Rice Genomes Project (3K RGP) (3K RGP 2014), researchers recently used Illumina next-generation technology to sequence a core collection of 3024 rice accessions from 89 countries. Consequently, sequence data with a high coverage (approximately 94%) and mapping rate (approximately 92.5%) were generated for the construction of a high-density SNP database (Alexandrov et al. 2015), thereby providing genotype data for a GWAS of rice agronomic traits. Considerable genetic diversity regarding SNPs, structural variations, and gene presence/absence variations has been revealed among these cultivated rice accessions (Wang et al. 2018).

In the present study, 563 rice accessions, mainly belonging to *Xian* (also known as *Indica*), *Geng* (also known as *Japonica*), and *Aus* subgroups, from 47 countries and areas with similar heading dates as those in Beijing (China) were selected from the 3024 rice genomes sequenced by 3K RGP (3K RGP 2014; Wang et al. 2018). These accessions underwent an association analysis of their resistance to a representative *R. solani* strain from China. We used 2,977,750 SNPs filtered from the 3K RGP 4.8 mio SNP dataset in the Rice SNP-Seek Database (Alexandrov et al. 2015). The objectives of our study were as follows: (1) identify ShB resistance resources in rice germplasm; (2) identify loci and candidate genes related to ShB resistance; and (3) elucidate the genetic mechanism underlying the quantitative resistance to ShB in rice. The data presented herein may be useful for improving ShB resistance by marker-assisted selection in rice breeding programmes.

## Results

### Population Structure of Rice Accessions

**Fig. 1:**
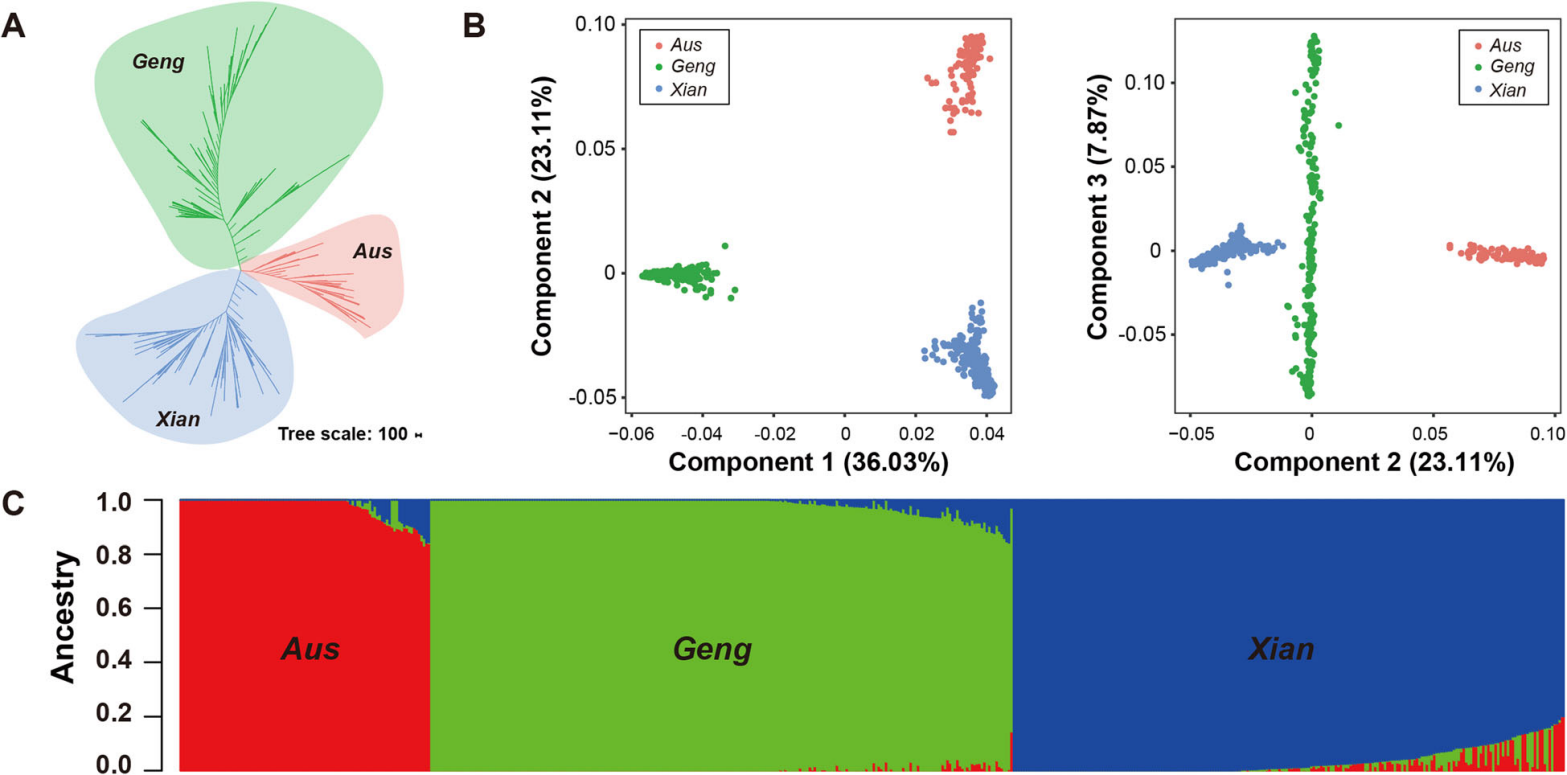
Population structure of 563 rice accessions. **a** Neighbour-joining tree constructed from LD-pruned SNPs. **b** Principal component analysis plots for the first three components. Left: first and second components; right: second and third components. **c** Distribution of the estimated subpopulation components (ancestry fraction) for each accession as determined by ADMIXTURE

A total of 220,335 independent SNPs with minor allele frequency (MAF) > 5% and missing data ratio (MDR) < 0.1 were used for genetic structure analyses. A neighbour-joining tree developed using PHYLIP (version 3.6) (Felsenstein 1989), with 100 bootstrap replicates, revealed that the 563 accessions could be classified into three main clades (Fig. 1a). A population structure analysis using ADMIXTURE (Alexander et al. 2009) (optimal number of subpopulations K = 3) also indicated that the 563 accessions belonged to three distinct clusters (Fig. 1c). Similar results were observed for the principal component analysis with 67.02% of the genetic variation in the accessions explained by the first three principal components. When we plotted the first three components against each other, most accessions were clustered in three groups (Fig. 1b). Thus, the 563 rice accessions were classified into the following three well-known subgroups: *Xian* (224 accessions), *Geng* (237 accessions), and *Aus* (102 accessions) (Additional file 1: Table S1). These results suggested that the accessions used as a covariate within the GWAS model in this study exhibited an obvious subpopulation structure.

### Evaluation of the Resistance of Rice to *R. solani*

**Fig. 2:**
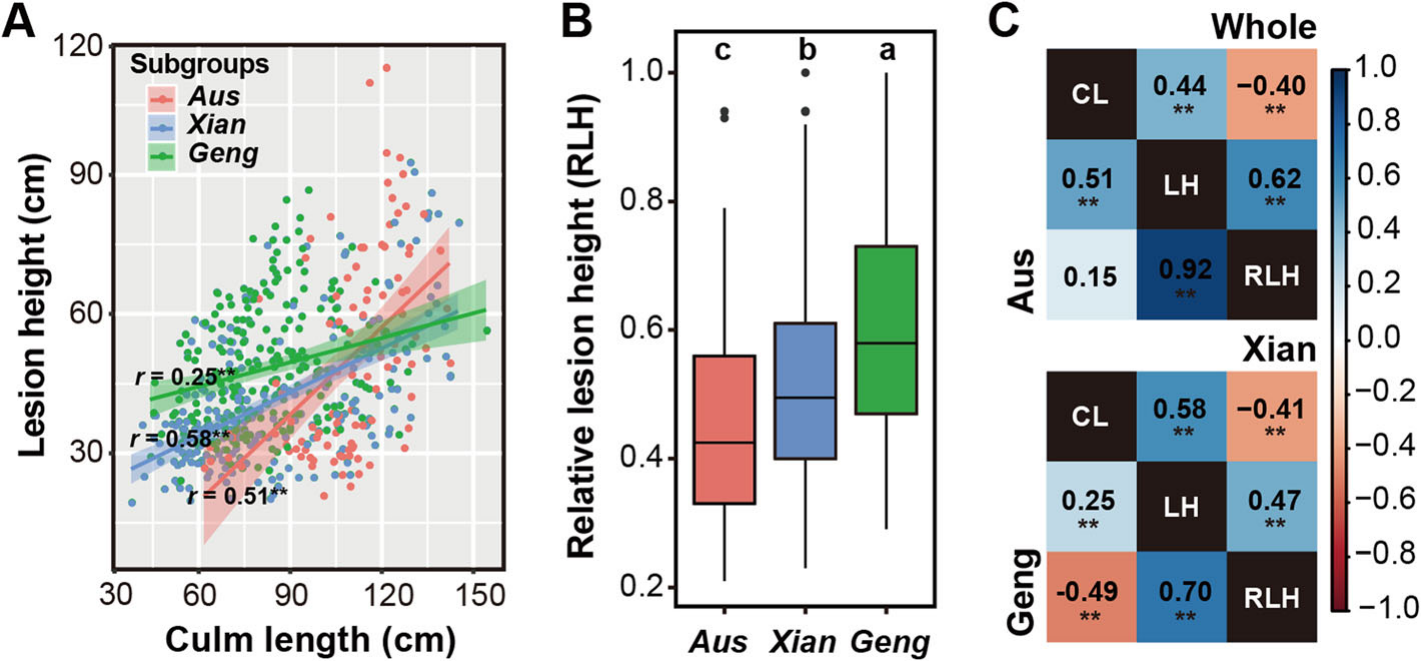
Evaluation of the traits related to ShB resistance and correlations among traits observed in different rice subgroups. **a** Dot plot of correlations between culm length (CL) and lesion height (LH). Orange, blue, and green dots represent *Aus*, *Xian*, and *Geng* accessions, respectively. Orange, blue, and green trend lines and shadowing represent linear regression lines and confidence intervals, respectively, between CL and LH within *Aus*, *Xian*, and *Geng* subgroups. **b** Relative lesion height (RLH) in different subgroups. Different characters indicate significant differences between rice subgroups (*P* < 0.01). **c** Correlations among CL, LH, and RLH from each GWAS panel. The number in the middle of the cell is the correlation coefficient and ‘**’ refers to a significant correlation (*P* < 0.01)

An assessment of the resistance of the 563 tested accessions to *R. solani* based on the relative lesion height (RLH) revealed considerable variations within and between subgroups (Fig. 2). Moreover, 39 accessions with an RLH of 0.21–0.30 were considered to exhibit moderate resistance to *R. solani* based on the Standard Evaluation for rice (IRRI 2002). These accessions corresponded to 22 *Aus* and 17 *Xian* accessions (Additional file 1: Table S1). A dot plot indicated the lesion height (LH) was positively correlated with culm length (CL), but the correlation in *Aus* was much stronger than that in *Geng*, suggesting a strong differentiation in the resistance to *R. solani* among the three rice subgroups (Fig. 2a). The results of an analysis of variance demonstrated that the mean RLH and CL were significantly lower and higher in the *Aus* subgroup than in the *Geng* and *Xian* subgroups, respectively. Additionally, the mean LH in the *Xian* subgroup was significantly lower than that in the *Aus* and *Geng* subgroups (Additional files 2 and 3: Tables S2 and S3). The average RLH values for the *Aus*, *Xian*, and *Geng* subgroups were 0.46, 0.52, and 0.61, respectively. Multiple comparisons revealed that the lowest RLH value was associated with the *Aus* subgroup (Fig. 2b), implying that the *Aus* accessions were more resistant to ShB than the accessions in the other two subgroups. The results of a correlation analysis among CL, LH, and RLH values across the whole panel and three subgroups are presented in Fig. 2c. There were significant positive correlations between LH and RLH in the whole panel and three subgroups, with the strongest correlation (*r* = 0.92, *P* < 0.01) detected in the *Aus* subgroup (Fig. 2c). Interestingly, there were significant negative correlations between CL and RLH in the whole panel as well as the *Xian* and *Geng* subgroups, but not in the *Aus* subgroup (Fig. 2c).

### Genome-Wide Association Signals for Sheath Blight Resistance in Rice

**Fig. 3:**
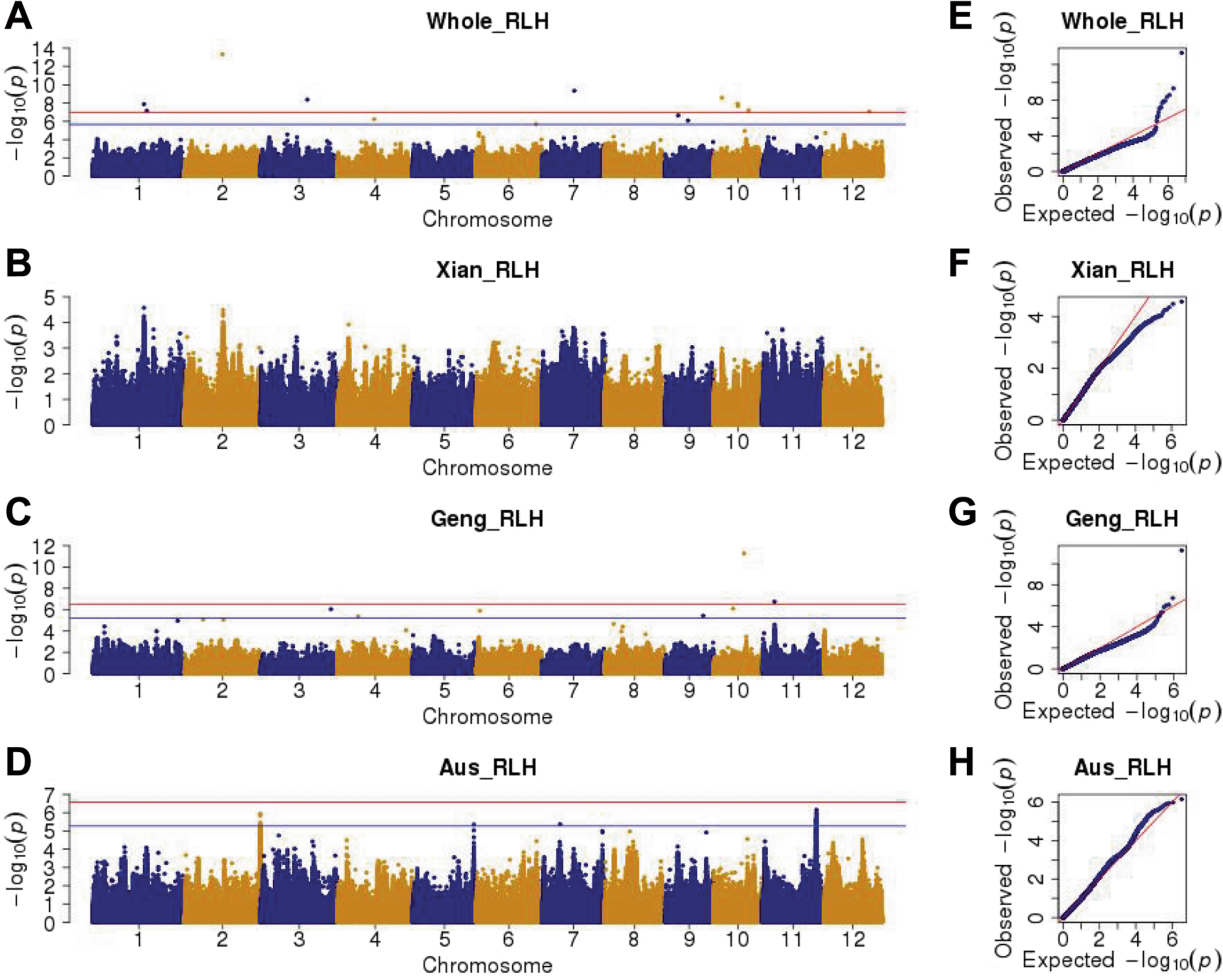
Manhattan and quantile-quantile plots for relative lesion height (RLH) for the whole, *Xian*, *Geng*, and *Aus* association panels based on the multi-locus GWAS method of FarmCPU. **a**, **e** Whole association panel. **b**, **f** *Xian* association panel. **c**, **g** *Geng* association panel. **d**, **h** *Aus* association panel. The strength of the associations for the RLH is indicated as the negative logarithm of the *P* value. Horizontal blue lines in the Manhattan plots indicate the following genome-wide suggestive thresholds (*P* values adjusted by a Bonferroni correction based on the effective number of independent markers calculated using GEC software): 2.16 × 10^− 6^, 3.28 × 10^− 6^, 6.57 × 10^− 6^, and 5.34 × 10^− 6^ for the whole, *Xian*, *Geng*, and *Aus* association panels, respectively. Horizontal red lines in the Manhattan plots indicate the following genome-wide significance thresholds (*P* < 0.05): 1.08 × 10^− 7^, 1.64 × 10^− 7^, 6.57 × 10^− 6^, and 2.67 × 10^− 7^ for the whole, *Xian*, *Geng*, and *Aus* association panels, respectively

We conducted association studies to identify genome-wide associated signals underlying the quantitative resistance to *R. solani* in the whole, *Aus*, *Xian*, and *Geng* panels to minimize the impact of rice population structure on the detection power of the GWAS. A total of 2,977,750 (whole), 1,665,543 (*Aus*), 1,776,496 (*Xian*), and 1,314,743 (*Geng*) SNPs with MAF > 5% and MDR < 0.1 were used for the association analyses according to the single-locus linear mixed model (LMM) by the Efficient Mixed Model Association eXpedited (EMMAX) and the multi-locus LMM by a fixed and random model with a circulating probability unification (FarmCPU) (Additional files 10, 11, 12, 13, 14: Figures S1–S5 and Fig. 3). Additionally, on the basis of a Bonferroni correction involving the effective number of independent markers at a significance level of 0.05 (Li et al. 2012), the genome-wide suggestive thresholds were *P* = 2.16 × 10^− 6^, 3.28 × 10^− 6^, 6.57 × 10^− 6^, and 5.34 × 10^− 6^ for the whole, *Xian*, *Geng*, and *Aus* panels, respectively (Additional file 4: Table S4). A total of 562 and 209 suggestive association signals were detected in one or more of the panel populations by EMMAX and FarmCPU, respectively (Additional file 5: Table S5). We identified 676 suggestively associated SNPs with different physical positions, namely quantitative trait nucleotides (QTNs), in at least one of the GWAS panels by EMMAX or FarmCPU, including 132, 498, and 62 QTNs for CL, LH, and RLH, respectively (Additional file 5: Table S5). Of the 676 QTNs, 553 SNPs either produced a synonymous substitution or were located in intergenic regions and introns. According to the reference Nipponbare genome IRGSP 1.0, the remaining SNPs were associated with large effects, and were detected in promoters (57), missense variants (32), 5′ UTRs (9), 3′ UTRs (14), start codons (1), splice regions (4), and stop codons (6) (Additional file 5: Table S5). Details regarding these suggestive association signals are listed in Additional file 5: Table S5. The average physical distance between neighbouring SNPs was 125.2, 223.8, 209.9, and 283.5 bp in the whole, *Aus*, *Xian*, and *Geng* panels, respectively. The average estimated linkage disequilibrium (LD) block region for all 12 chromosomes in the whole panel was 20.6 kb, ranging from 12.1 kb on chromosome 11 to 50.7 kb on chromosome 3. Thus, we combined adjacent QTNs within an LD block to form a suggestively associated locus (SAL) for ShB resistance in each GWAS panel. Moreover, the QTN with the minimum *P* value in a cluster was considered to be the lead SNP. We focused on the identified RLH associations due to the major parameter used to assess the ShB resistance in this study (Fig. 3 and Additional file 14: Figure S5).

A total of 27 SALs for ShB resistance containing 62 suggestive QTNs (including 11 SALs containing 12 significant QTNs) were detected on all chromosomes except for chromosome 8 by merging the QTNs based on EMMAX or FarmCPU in all panels (Table 1). Remarkably, significant QTNs for RLH were not identified in the *Aus* and *Xian* panels, and no QTN was detected in more than one GWAS panel. At least 12 SALs (L4, L7, L10, L13, L15, L16, L18, L19, L23, L25, L26, and L27) were adjacent to one or more previously characterized ShB resistance-related QTLs/genes. In the whole panel, L3 (17.85–17.96 Mb) on chromosome 2 and L15 (15.24–15.25 Mb) on chromosome 7, had the most significant association signals for RLH. We also determined that another previously reported ShB resistance QTL (*qSBR7–1*) in Tetep (Channamallikarjuna et al. 2010) was located nearby the L15 region. Notably, no QTL/gene related to ShB resistance was reported in the L3 region (17.85–17.96 Mb) on chromosome 2, which included the most significant association signal (rs2_17,889,517, *P* = 4.8 × 10^− 14^) in the whole panel, suggesting this region may contain a potentially novel SAL that should be fine-mapped (Table 1). In the *Geng* panel, L23 (14.42–14.59 Mb) on chromosome 10 and L25 (5.70–5.77 Mb) on chromosome 11 were the only two SALs related to significant association signals. In the *Aus* panel, there were two SALs localized to two hotspot regions, namely L4 on chromosome 2 and L26 on chromosome 11, detected for RLH and LH by EMMAX and FarmCPU. The lead SNP (rs11_25,580,510, *P* = 7.0 × 10^− 7^) of L26 was located in the intergenic region between *LOC_Os11g42450* and *LOC_Os11g42470*, which encode an LRR family protein and a protein with an unknown function, respectively. In the *Xian* panel, only SAL L5 (16.20–16.25 Mb) on chromosome 3 was detected. Similar to L3, L5 was not associated with previously reported ShB resistance QTLs/genes. The lead SNP (rs3_16,214,232, *P* = 9.5 × 10^− 7^) of L5 was detected in the promoter region of a gene associated with an unknown function (*LOC_Os03g28170*).

**Table 1:** Significantly associated loci for sheath blight resistance in rice based on a genome-wide association study

SAL ID	Chr.	LD block interval (bp)	LD block size (kb)	No. of suggestive SNPs in LD block	Panel	Lead SNP position (bp)	Lead SNP *P* value	MSU ID of genes harboring lead SNP	QTL/Marker/Gene previously reported
L1	1	23,999,888–23,999,975	0.1	1	Whole	23,999,975	1.3E-08	LOC_Os01g42294-LOC_Os01g42310	
L2	1	25,337,514–25,449,724	112.2	1	Whole	25,350,468	6.8E-08	LOC_Os01g44200-LOC_Os01g44210	
L3	2	17,849,943–17,960,809	110.9	1	Whole	17,889,517	4.8E-14	LOC_Os02g30114	
L4	2	35,718,082–35,816,634	98.6	17	*Aus*	35,752,298	8.9E-07	LOC_Os02g58470	*QDs2b* and *QRh2b* (Li et al. 2009)
L5	3	16,203,266–16,251,439	48.2	1	*Xian*	16,214,232	9.5E-07	LOC_Os03g28170	
L6	3	21,967,065–22,099,455	132.4	1	Whole	22,062,214	4.2E-09	LOC_Os03g39660-LOC_Os03g39670	
L7	3	33,180,575–33,294,172	113.6	1	*Geng*	33,211,402	9.3E-07	LOC_Os03g58290-LOC_Os03g58300	*qSBR-3* (Kunihiro et al. 2002); *qSBPL-3-2* (Wen et al. 2015); *qHNLH3* (Zeng et al. 2015); *OsWRKY4* (Wang et al. 2015)
L8	4	9,578,771–9,768,308	189.5	1	*Geng*	9,676,399	4.5E-06	LOC_Os04g17660-LOC_Os04g17669	
L9	4	17,248,318–17,355,392	107.1		Whole	17,255,541	5.8E-07	LOC_Os04g29080-LOC_Os04g29090	
L10	5	28,879,416–28,948,601	69.2	1	*Aus*	28,939,653	4.4E-06	LOC_Os05g50480	RM5784 and RM3286 (Taguchi-Shiobara et al. 2013)
L11	6	1,826,635–1,964,451	137.8	1	*Geng*	1,913,825	1.3E-06	LOC_Os06g04460	
L12	6	22,541,170–22,604,225	63.1	1	Whole	22,546,226	2.0E-06	LOC_Os06g38100-LOC_Os06g38110	
L13	6	28,352,324–28,363,626	11.3	1	Whole	28,362,652	2.1E-06	LOC_Os06g46680-LOC_Os06g46690	*QRlh6* (Xie et al. 2008); *qshb6.1* (Yadav et al. 2015); *OsCHI11* (Karmakar et al. 2017)
L14	7	8,485,551–8,669,352	183.8	1	*Aus*	8,569,372	4.2E-06	LOC_Os07g14940-LOC_Os07g14950	
L15	7	15,236,665–15,247,698	11.0	1	Whole	15,244,300	4.4E-10	LOC_Os07g26490-LOC_Os07g26500	*qSBR7–1* (Channamallikarjuna et al. 2010)
L16	9	6,313,772–6,328,962	15.2	1	Whole	6,317,362	2.3E-07	LOC_Os09g11360-LOC_Os09g11370	*qHZaDR9* (Zeng et al. 2015)
L17	9	10,897,450–10,929,683	32.2	1	Whole	10,915,359	8.2E-07	LOC_Os09g17830-LOC_Os09g17840	
L18	9	18,082,609–18,092,891	10.3	1	*Geng*	18,092,604	3.9E-06	LOC_Os09g29760-LOC_Os09g29780	*qSB-9* (Han et al. 2002); *qSBR9–1* (Channamallikarjuna et al. 2010); *qshb9.1* (Yadav et al. 2015); RM6251 and RM3533 (Taguchi-Shiobara et al. 2013); bc15/OsCTL1 (Wu et al. 2012)
L19	10	3,996,721–4,021,580	24.9	1	Whole	4,011,130	2.5E-09	LOC_Os10g07520-LOC_Os10g07530	*QRlh10b* (Xie et al. 2008)
L20	10	9,226,748–9,327,350	100.6	1	*Geng*	9,326,435	8.1E-07	LOC_Os10g18360	
L21	10	11,281,341–11,387,999	106.7	1	Whole	11,381,173	1.2E-08	LOC_Os10g22030	
L22	10	11,610,905–11,623,071	12.2	1	Whole	11,620,213	2.1E-08	LOC_Os10g22440-LOC_Os10g22450	
L23	10	14,423,809–14,585,826	162.0	1	*Geng*	14,523,517	5.3E-12	LOC_Os10g28009-LOC_Os10g28020	*qDs10* (Li et al. 2009)
L24	10	16,612,339–16,716,107	103.8	1	Whole	16,616,152	6.1E-08	LOC_Os10g31680-LOC_Os10g31700	
L25	11	5,702,715–5,767,077	64.4	1	*Geng*	5,708,512	1.8E-07	LOC_Os11g10460-LOC_Os11g10470	*qSB-11*^*LE*^ (Zuo et al. 2013); *qSBPL-11-2* (Wen et al. 2015)
L26	11	25,530,571–25,595,331	64.8	33	*Aus*	25,580,510	6.9E-07	LOC_Os11g42450-LOC_Os11g42470	*QDs11b* and *QRh11* (Li et al. 2009); *qSBR11–1* (Channamallikarjuna et al. 2010)
L27	12	21,483,436–21,483,932	0.5	1	Whole	21,483,904	8.4E-08	LOC_Os12g35340-LOC_Os12g35350	*qSBPL-12* (Wen et al. 2015)

### Identifying Putative Functional Genes Associated with Sheath Blight Resistance

For a given GWAS locus, the gene nearest to the lead SNP is not always the causal gene (Brodie et al. 2016). Therefore, all genes located in the LD blocks of detected SALs underwent an extensive haplotype analysis to identify putative functional genes. The LD blocks of all detected SALs except for L1 and L27 included more than one gene annotated based on the Nipponbare reference genome IRGSP 1.0. A total of 316 genes were detected in 25 LD blocks (L2–L26). Of these genes, 283 with at least one SNP in the whole GWAS panel were included in a haplotype analysis (e.g., haplotype frequency in each subpopulation) and multiple comparison tests of the RLH in each GWAS panel. Consequently, 261 ShB resistance putative functional genes (SRPFGs) were identified in all SALs except for L1, L12, L13, and L27, with significant differences in the RLH among different haplotypes in at least one GWAS panel. These 261 genes comprised 116 functionally annotated genes, 57 transposons, and 88 genes with unknown functions (Additional file 6: Table S6). Our findings may be useful for identifying the genes responsible for ShB resistance. The number of SRPFGs per SAL ranged from 1 in L22 to 25 in L23, with a mean of 11.3 ± 7.3. These genes were significantly enriched in the gene ontology (GO) biological processes related to plant cellular amino acid metabolic processes (Additional file 7: Table S7). Moreover, they were also significantly enriched in plant metabolic pathways, including fatty acid biosynthesis and degradation based on the Kyoto Encyclopedia of Genes and Genomes (KEGG) pathway database (Additional file 8: Table S8). Out of the 204 non-transposon SRPFGs, we detail showed haplotype analyses of 36 genes within 20 detected SALs (Additional file 9: Table S9) if it met any of the following conditions: (i) involving in GO classification of response to stress term; (ii) genes with known function on disease resistance; (iii) as one hit gene in the significantly enriched pathways, and (iv) the relatively lower *p*-value of the haplotype analyses in Additional file 6: Table S6. Two of these genes (*LOC_Os10g28050* and *LOC_Os06g04510*) were subsequently analysed as follows.

**Fig. 4:**
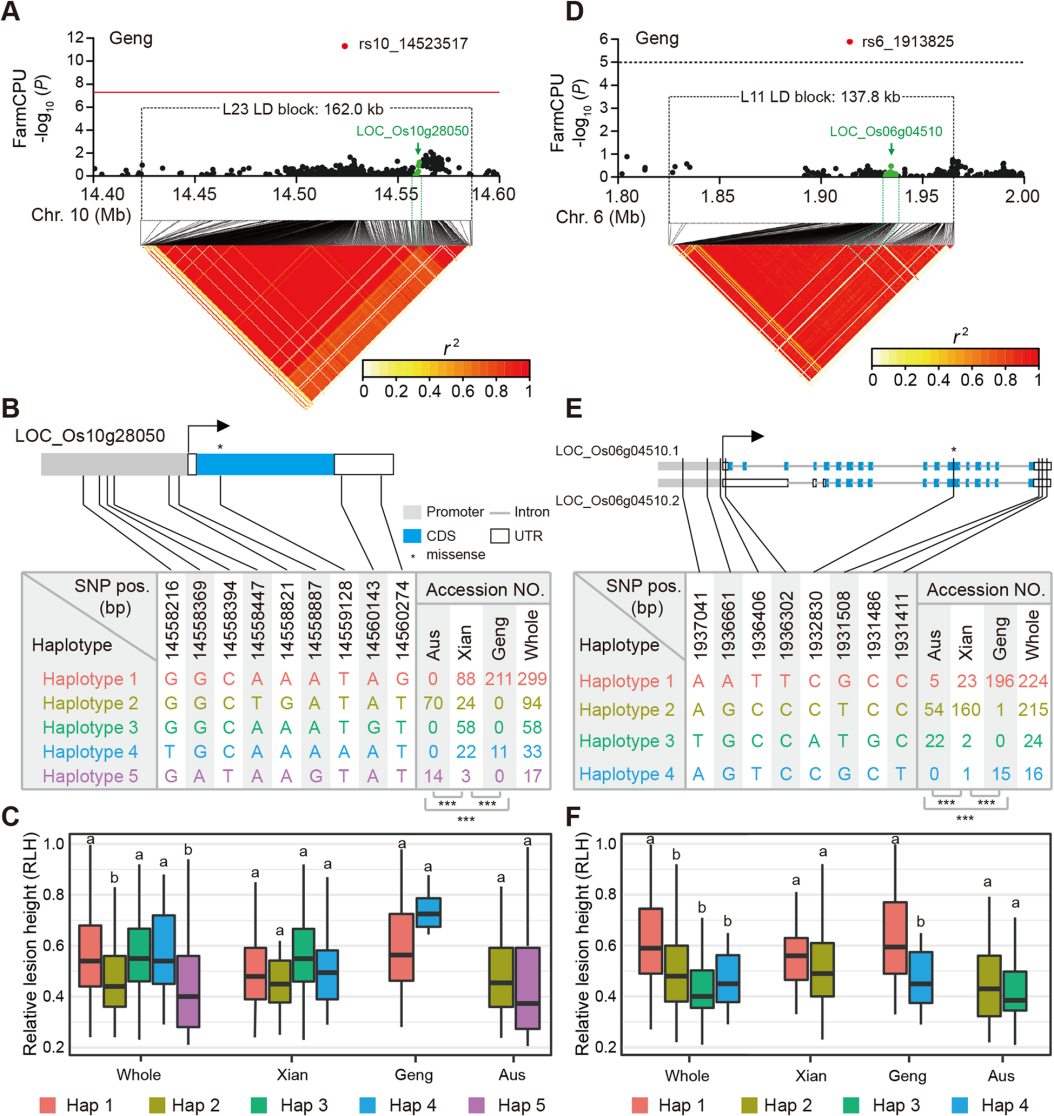
Associated regions for relative lesion height (RLH) in the *Geng* panel and a haplotype analysis of the candidate genes within the associated regions on chromosomes 6 and 10. Local Manhattan plots (top) and linkage disequilibrium heatmaps (bottom) surrounding the hotspot regions on chromosomes 10 (**a**) and 6 (**d**). Red dots indicate the position of the peak SNPs identified by FarmCPU, while green arrows and dots indicate the position of the candidate genes within the LD blocks. Gene structure and haplotype analysis of the sheath blight resistance candidate genes *LOC_Os10g28050* (**b**) and *LOC_Os06g04510* (**e**). Haplotypes with fewer than 10 accessions are not shown. Relative lesion height of accessions in different haplotypes of *LOC_Os10g28050* (**c**) and *LOC_Os06g04510* (**f**) in different association panels. Characters on top of boxplots indicate significant differences based on Duncan’s multiple comparison tests (*P* < 0.05)

We detected *LOC_Os10g28050*, which encodes a chitinase 2, in the LD block region of L23. This gene was located 34 kb downstream of the lead SNP rs10_14,523,517 (Fig. 4a). The haplotypes were built based on six SNPs in a 1-kb region upstream of the *LOC_Os10g28050* promoter, one SNP in the coding region with a missense mutation, and two SNPs in the 3′ UTR (Fig. 4b). We detected five haplotypes shared by at least 10 accessions in 501 of 563 accessions (Fig. 4b and Additional file 9: Table S9). A comparison of the RLHs for the five haplotypes revealed that accessions Hap2 and Hap5 had significantly lower RLHs than the other three haplotypes (*P* < 0.01; Fig. 4c and Additional file 9: Table S9). Furthermore, in the *Aus* panel, Hap2 was represented by 70 (74.5%) of 94 accessions, while 14 (82.4%) of 17 accessions corresponded to Hap5. In contrast, the *Geng* panel lacked Hap2 and Hap5 accessions (Fig. 4c and Additional file 9: Table S9). These results partially explained the variability in the resistance to *R. solani* observed among the *Aus*, *Xian*, and *Geng* panels.

We located *LOC_Os06g04510*, which encodes an enolase, in the LD block region of L11. This gene was located 17 kb downstream of the lead SNP rs6_1,913,825 (Fig. 4d). According to the Nipponbare reference genome IRGSP 1.0, *LOC_Os06g04510* is associated with two transcripts. We analysed these two transcripts, and identified four major haplotypes shared by at least 10 of 479 accessions based on eight SNPs in a 1-kb upstream region, gene coding region, and the 5′ and 3′ UTRs (Fig. 4e). We compared the mean RLH among those haplotypes in each GWAS panel. The RLH of Hap1 was significantly higher than that of the other three haplotypes in the whole panel. Notably, in the *Geng* panel, the RLH of Hap4 was significantly lower than that of Hap1 (*P* = 0.0045) (Fig. 4f and Additional file 9: Table S9). Among the 224 accessions with the Hap1 major haplotype, 196 (87.5%) accessions belonged to the *Geng* panel. Additionally, all but one of the Hap2 accessions were from the *Xian* and *Aus* panels, with the exception belonging to the *Geng* panel. Of the two minor haplotypes, 91.7% of Hap3 accessions belonged to the *Aus* panel, while 93.8% of Hap4 accessions belonged to the *Geng* panel. These results were useful for clarifying the phenotypic variability within the *Geng* panel in response to *R. solani*.

## Discussion

During the past 3 decades, considerable efforts have been made to screen rice germplasm for ShB resistance (Srinivasachary and Savary 2011; Yadav et al. 2015). However, no cultivars with complete resistance are available and no major genes conferring immunity to *R. solani* have been identified. Most QTLs conferring ShB resistance identified using bi-parental linkage mapping populations exhibited small effects (Zeng et al. 2011). A GWAS based on diversified germplasm with high-density genotypes may provide new insights into the genetic basis of agronomic traits in crops. In the present study, we conducted a large-scale GWAS based on a rice core collection and approximately 3 million SNPs to dissect the genetic mechanisms underlying quantitative resistance to *R. solani* and to identify novel loci and alleles associated with resistance. Our results may provide useful information for improving ShB resistance in rice.

### Diverse Reactions of Accessions to *Rhizoctonia solani* among Rice Subgroups

In this study, multiple comparisons of RLH among three panels revealed *Aus* accessions were the most resistant to ShB, followed by the *Xian* and *Geng* accessions (Fig. 2b), which is consistent with the results of previous studies (Chen et al. 2019; Willocquet et al. 2012; Zuo et al. 2008). The data from bi-parental mapping populations in earlier investigations implied that RLH is negatively correlated with plant height or CL (Li et al. 2009; Li et al. 1995; Wen et al. 2015), and seedling height and disease score of ShB resistance is aslo negatively correlated in rice diversity panel 1 (Chen et al. 2019). Our results also showed significantly negative correlations between RLH and CL in *Xian* and *Geng* accessions (Fig. 2c). Therefore, CL significantly affected the evaluation of the ShB resistance based on RLH. However, there was a non-significant correlation between RLH and CL in the *Aus* accessions (Fig. 2c), indicating that the influence of CL on evaluation of ShB resistance can be effectively eliminated in *Aus* panel. One possible reason for the differing relationship between CL and RLH in *Aus* compared with *Xian* and *Geng* is the relatively large variation of LH versus small of variation of CL in *Aus* (Additional file 4: Table S3), which explains the Type II SALs L4 and L26 could be detected for both RLH and LH (Additional file 5: Table S5). We further examined the relationships between the RLH- and CL-SALs. Only one co-localization of CL-SAL and RLH-SAL L5 at approximately 16.2 Mb on chromosome 3 was detected in the *Xian* panel (Additional file 5: Table S5). These results suggested that most of the RLH-SALs identified in this study were more likely to control the molecular mechanisms underlying physiological resistance mentioned by Srinivasachary et al. (Srinivasachary and Savary 2011) rather than the disease escape strongly determined by plant architecture. These findings may be useful for enhancing ShB resistance in rice cultivars.

### Comparison of SAL and QTL Mapping for Sheath Blight Resistance

According to our results, association studies involving a combination of a single locus genome-wide scan (EMMAX) and a multi-locus GWAS method (FarmCPU) are more efficient in determining the number of loci controlling rice quantitative resistance to *R. solani* than classical bi-parental linkage mapping methods. One possible explanation is that only QTLs with segregating alleles between limited founder parents can be identified by traditional QTL linkage mapping. We observed that 12 (44.4%) of 27 GWAS loci associated with RLH harboured multiple previously reported QTLs/genes related to ShB resistance (Table 1). For example, in the chromosomal region near SAL L25, one well-known major QTL (*qSB-11*^LE^) for ShB resistance was fine-mapped using a set of chromosome segment substitution lines in the Lemont genetic background with Teqing as a donor parent (Zuo et al. 2013). Meanwhile, another QTL (*qSBPL-11-2*) was detected using three Lemont/Yangdao4 mapping populations (Wen et al. 2015) **(**Table 1 and Additional file 5: Table S5). Additionally, SAL L23, with the most significant SNP (rs10_14,523,517, *P* = 5.3 × 10^− 12^) associated with RLH in the *Geng* panel, contained one QTL (*qDs10*) for disease severity in a Teqing/Binam backcross introgression line (BIL) population (Li et al. 2009) (Table 1 and Additional file 5: Table S5). Moreover, two previously reported QTLs (*QDs2b* and *QRh2b*) (Li et al. 2009) identified in a Teqing/Tarom Molaii BIL population were found adjacent to the L4 region (35.72–35.82 Mb). Moreover, SAL L26 included 33 suggestively associated SNPs spanning an approximately 64.8-kb interval (25.53–25.60 Mb) on chromosome 11, with another two reported ShB resistance QTLs (*QDs11b* and *QRh11*) from a Teqing/Tarom Molaii BIL population (Li et al. 2009) and one fine-mapped QTL (*qSBR11–1*) from rice line Tetep with a high degree of resistance to *R. solani* (Channamallikarjuna et al. 2010) (Table 1 and Additional file 5: Table S5). The co-localization of known QTLs/genes helped to verify the SALs identified in our study. These confirmed SALs represent candidates for fine-mapping, gene cloning, and marker-assisted selection for improving the ShB resistance of rice cultivars.

### Putative Defence Mechanism against Sheath Blight in Rice

The molecular mechanisms involved in the host–pathogen interactions underlying quantitative ShB resistance in rice remain unclear. Some studies concluded that a jasmonic acid (JA)-induced resistance pathway (Karmakar et al. 2017; Wang et al. 2015) and a salicylic acid (SA)-mediated systemic acquired resistance pathway (Kouzai et al. 2018; Molla et al. 2016) might influence the resistance of rice to *R. solani*. These two important pathways, which form part of the defence system in rice, have common defence-related and pathogenesis-related proteins such as chitinases (Datta et al. 2001; Karmakar et al. 2016; Karmakar et al. 2017; Richa et al. 2017), glucanases (Datta et al. 1999), and OsWRKY transcription factors (Wang et al. 2015). Chitinase production can be induced by JA in rice (Rakwal et al. 2004). One chitinase gene, *LOC_Os11g47510*, was recently cloned from a QTL region (*qSBR11–1*) for ShB resistance in the *R. solani*-tolerant rice line Tetep, and subsequently validated by a genetic transformation into susceptible rice line Taipei 309 (Richa et al. 2017). The overexpression of several other chitinase genes, such as *OsCHI11* and *RCH10*, have resulted in increased ShB tolerance in transgenic plants (Lin et al. 1995; Mao et al. 2014).

The involvement of SA, which is a product of phenylpropanoid metabolism (Lee et al. 1995), in the resistance of rice to *R. solani* was confirmed in SA-deficient transgenic plants (Kouzai et al. 2018). The glycolytic pathway is reportedly important for defence responses against *R. solani* and is connected to the phenylpropanoid pathway (Mutuku and Nose 2012). Additionally, SA biosynthesis may be indirectly induced by cuticular wax accumulation during resistance responses in plants (Kouzai et al. 2018; Seo et al. 2011). Cuticular wax is mainly composed of long-chain aliphatic compounds, and wax biosynthesis in plants begins with the synthesis of fatty acids in the plastid (Kunst and Samuels 2003). The wax forms a natural barrier against biotic and abiotic stresses during plant growth and development (Wang et al. 2017). In rice cultivars, the amount of cuticular wax deposits on the outer sheaths are negatively correlated with the infection rates and formation of infection cushions by *R. solani* (Marshall and Rush 1980).

In the present study, 36 ShB resistance genes were screened from 261 SRPFGs within LD blocks of 23 RLH-SALs based on the annotation of gene functions, enriched KEGG pathways and GO terms, and multiple comparisons among haplotypes (Additional file 9: Table S9). Our data suggest that JA and SA signalling pathways might regulate rice responses to ShB. For example, *LOC_Os10g28050*, which encodes chitinase 2, was identified 34 kb downstream of the lead SNP rs10_14,523,517 of the L23 LD block (Fig. 4a). The mean RLHs of the Hap2 and Hap5 accessions carrying *LOC_Os10g28050* were significantly lower than that of the other three haplotypes. The resistance haplotypes (Hap2 and Hap5) were mainly enriched in the *Aus* panel, and the five haplotypes also exhibited a distribution tendency among subgroups (Fig. 4c and Additional file 9: Table S9). Additionally, *LOC_Os06g04510*, which encodes an enolase that participates in the glycolytic pathway, was detected 17 kb downstream of the lead SNP rs6_1,913,825 of SAL L11 (Fig. 4d). The mean RLH of Hap3 accessions with this gene was significantly lower than that of the other three haplotypes. Similar to *LOC_Os10g28050*, the *LOC_Os06g04510* resistance haplotype of Hap3 accessions was mainly enriched in the *Aus* panel. Furthermore, two haplotypes (Hap1 and Hap4) explained the phenotypic variability within the *Geng* panels in response to *R. solani* (Fig. 4c and Additional file 9: Table S9).

In this study, significant differences in the mean RLH among different haplotypes within subgroups or the whole population were identified for two genes (*LOC_Os02g30060* and *LOC*_*Os10g31780*) involved in fatty acid biosynthesis and one gene (*LOC_Os11g10520* encoding a dehydrogenase) related to the degradation of aromatic compounds (Additional files 8 and 9: Tables S8 and S9). Molecular studies have revealed that rice plants expressing the gene encoding OsGL1–1, which contains regions homologous to parts of short-chain dehydrogenases, exhibit induced deposition of cuticular wax in contrast to the *osgl1–1* mutant lacking the corresponding gene (Qin et al. 2011).

Transient expression assays revealed that SA-inducible OsWRKY6 is a positive regulator of a constitutively activated pathogenesis-related gene (*OsPR10a*), and *OsWRKY6*-overexpressing transgenic rice plants exhibit enhanced resistance to pathogens (Choi et al. 2015). In this study, we determined that *OsWRKY6* (*LOC_Os03g58420*), located in SAL L7 near three previously mapped QTL regions (*qSBR-3*, *qSBPL-3-2*, and *qHNLH3*) is associated with ShB resistance (Additional file 9: Table S9). Regarding this gene, multiple comparisons of the RLH revealed that Hap3 accessions were most resistant to ShB, followed by Hap2 and Hap1 accessions. Moreover, Hap3 was absent in the *Xian* and *Geng* accessions, but was relatively abundant in the *Aus* panel, confirming a strong differentiation among three rice subgroups. However, some OsWRKY transcription factors, such as OsWRKY4 and OsWRKY30, are reportedly important positive regulators of rice responses to ShB mediated by a JA-dependent signalling pathway (Peng et al. 2012; Wang et al. 2015). These results provide novel information regarding the genes involved in ShB resistance and further elucidate the molecular mechanism underlying rice resistance to ShB.

## Conclusions

The current GWAS of ShB resistance detected suggestive signals, estimated the candidate regions with suggestive signals based on LD blocks, and predicted the causal genes according to bioinformatics and haplotype analyses. Our results imply that quantitative resistance to *R. solani* in rice may be mediated by SA- and JA-dependent signalling pathways. Our future research will focus on the functional validation of the identified candidate genes by genetic transformations and transcriptomics-based investigations. The findings reported herein may be useful for improving the application of rice germplasm resources as well as the knowledge-based management of ShB and the breeding of ShB-resistant rice cultivars.

## Methods

### Rice Germplasm and Evaluation of Sheath Blight Resistance under Field Conditions

We selected a diverse collection of 563 *Oryza sativa* accessions from 47 countries and areas without distinct unfavourable agronomic traits and with similar heading dates as those in Beijing, China (116°20′E, 40°22′N) from the 3K RGP database (3K RGP 2014) (Additional file 1: Table S1). To evaluate ShB resistance, the seeds of all rice accessions were sown in a seedling nursery, and 30-day-old seedlings were transplanted to the experimental farm at the Institute of Crop Sciences, Chinese Academy of Agricultural Sciences, Beijing, China. Each row (20 × 17 cm) comprised nine plants, which were inoculated with the highly pathogenic *R. solani* strain RH-9 at the late tillering stage as described by Zou et al. (Zou et al. 2000). Three central plants for each line were inoculated (with two replicates), with the third leaf sheath of the main stem and the four largest tillers of each plant inserted into the inoculum without changing the holding status of the sheath and stem (Zuo et al. 2008). The ShB resistance of each inoculated plant was evaluated 30 days later (Zeng et al. 2015) according to the RLH, which was calculated as the ratio between LH and CL. The three tillers with the highest lesions were selected for each plant, and the LH was measured along the stem from the lowest to highest sites, while the CL was estimated from the soil surface to the panicle neck. The mean trait value of one accession was calculated based on three individual plants (i.e., three lesions per plant) for each replicate. The average trait values of two replicates for each accession were used for the GWAS.

### Population Structure Analysis

The 3K RGP 4.8 mio SNP dataset was downloaded from the Rice SNP-Seek Database. http://snp-seek.irri.org/ (Alexandrov et al. 2015). To avoid the influence of linked SNPs during the population structure analysis, we used the LD pruning tool of the PLINK program (version 1.9) (Purcell et al. 2007) to obtain a subset of 220,335 independent SNPs with a MAF > 5% and a MDR < 0.1 according to ‘indep-pairwise 50 10 0.5’. We used PHYLIP (version 3.6) (Felsenstein 1989) to construct an unrooted neighbour-joining tree with 100 bootstrap replicates. The genetic structure of the whole population was predicted with the ADMIXTURE program (Alexander et al. 2009). Meanwhile, PLINK (Purcell et al. 2007) was used to conduct a principal component analysis to estimate the number of subpopulations in the GWAS panel.

### Genome-Wide Association Mapping

A total of 2,977,750 SNPs with a MAF > 5% and MDR < 0.1 were filtered for association analyses of the whole panel. The GWAS was completed using a LMM implemented in EMMAX program (Kang et al. 2010) as well as FarmCPU (Liu et al. 2016) to determine the associations between each SNP and three traits related to ShB resistance (LH, CL, and RLH). We used the Balding–Nichols matrix based on a pruned subset of 65,095 SNPs across the whole rice genome (with parameter ‘indep-pairwise 50 10 0.1’ in PLINK) to develop the kinship matrix, which measured the genetic similarity between individuals. The first three principal components were used as covariates (Q-matrix) to control for population structure. The effective number of independent markers (N) was calculated using GEC software (Li et al. 2012), and the suggestive and significant *P*-value thresholds of each GWAS panel were calculated (Additional file 4: Table S4). The Manhattan and quantile-quantile plots for the GWAS results were created using the R package qqman (Turner 2014). To obtain independent association signals, multiple suggestively associated SNPs located in one estimated LD block were clustered as one SAL, and the SNP with the minimum *P* value in a cluster was considered as the lead SNP. The LD block was estimated with the command ‘--blocks’ in PLINK according to the block definition suggested by Gabriel et al. (Gabriel et al. 2002). The pairwise LD *r*^2^ values within one estimated LD block were calculated with PLINK (Purcell et al. 2007), and the R package LDheatmap (Shin et al. 2006) was used to draw the heatmap of pairwise LDs.

### Annotation of Significant Signals

Synonymous and nonsynonymous SNPs and SNPs associated with large-effect changes were annotated based on the gene models of the annotated version of the Nipponbare reference genome IRGSP 1.0 (Kawahara et al. 2013) using the snpEff program (version 4.0) (Cingolani et al. 2012). Enriched GO terms and KEGG pathways were identified using the agriGO v2.0 (Tian et al. 2017) and EXPath 2.0 (Chien et al. 2015) programs, respectively. We have listed all suggestively associated SNPs located within genes and the annotation information based on the Nipponbare reference genome IRGSP 1.0 (Kawahara et al. 2013).

### Haplotype Analysis

The whole GWAS panel SNPs within 1 kb of the upstream promoter region, 3′ untranslated region (UTR), and 5′ UTR as well as non-synonymous SNPs in the coding regions of a candidate gene were concatenated as the haplotype. Only haplotypes shared by at least 10 accessions were used for multiple comparisons. For the multiple group comparison of the RLHs of the major haplotypes, Duncan’s multiple comparison tests followed by a one-way analysis of variance were completed with the agricolae package in R. Additionally, chi-square tests in R were used to determine significant differences in the frequency of different haplotypes for the candidate genes among rice subgroups.

## Supplementary information

**Additional file 1 : Table S1.** Summary of 563 rice accessions and their resistance to rice sheath blight.

**Additional file 2 : Table S2.** Analysis of the variance in traits related to sheath blight resistance.

**Additional file 3 : Table S3.** Multiple comparisons of traits related to sheath blight resistance among three rice subgroups.

**Additional file 4 : Table S4.** Filtered and effective number of single nucleotide polymorphisms across panels and adjusted significant *P* value thresholds based on a Bonferroni correction.

**Additional file 5 : Table S5.** Significant signals associated with resistance to *Rhizoctonia solani* detected in at least one of the rice panel populations based on two GWAS models.

**Additional file 6 : Table S6.** Sheath blight resistance putative functional genes for 23 RLH-SALs detected by a GWAS.

**Additional file 7 : Table S7.** Results of a GO enrichment analysis of sheath blight resistance putative functional genes.

**Additional file 8 : Table S8.** Results of a KEGG pathway enrichment analysis of sheath blight resistance putative functional genes.

**Additional file 9 : Table S9.** Haplotype analyses of 36 genes associated with RLH screened based on annotated functions and multiple comparisons.

**Additional file 10 : Figure S1.** Manhattan and quantile-quantile plots for culm length based on the whole, *Xian*, *Geng*, and *Aus* panels using EMMAX.

**Additional file 11 : Figure S2.** Manhattan and quantile-quantile plots for culm length based on the whole, *Xian*, *Geng*, and *Aus* panels using FarmCPU.

**Additional file 12 : Figure S3.** Manhattan and quantile-quantile plots for lesion height based on the whole, *Xian*, *Geng*, and *Aus* panels using EMMAX.

**Additional file 13 : Figure S4.** Manhattan and quantile-quantile plots for lesion height based on the whole, *Xian*, *Geng*, and *Aus* panels using FarmCPU.

**Additional file 14 : Figure S5.** Manhattan and quantile-quantile plots for relative lesion height based on the whole, *Xian*, *Geng*, and *Aus* panels using EMMAX.

## Data Availability

All data supporting the conclusions of this article are provided within the article (and its Additional files).

## Abbreviations

3K RGP: 3,000 Rice Genomes Project
BIL: Backcross introgression line
CL: Culm length
EMMAX: Efficient Mixed Model Association eXpedited
FarmCPU: Fixed and random model with a circulating probability unification
GO: Gene ontology
GWAS: Genome-wide association study
JA: Jasmonic acid
KEGG: Kyoto Encyclopedia of Genes and Genomes
LD: Linkage disequilibrium
LH: Lesion height
LMM: Linear mixed model
MAF: Minor allele frequency
MDR: Missing data ratio
QTL: Quantitative trait locus
QTNs: Quantitative trait nucleotides
*R.*: *solani*: *Rhizoctonia solani Kühn*
RLH: Relative lesion height
SA: Salicylic acid
SAL: Suggestively associated locus
ShB: Sheath blight
SNPs: Single nucleotide polymorphisms
SRPFGs: ShB resistance putative functional genes
