# Supplementary figures and images for: Genome-Wide Association Analysis of the Genetic Basis for Sheath Blight Resistance in Rice
Source: Rice (N Y). 2019 Dec 18;12:93. doi: 10.1186/s12284-019-0351-5 (PMC6920286; doi:10.1186/s12284-019-0351-5)

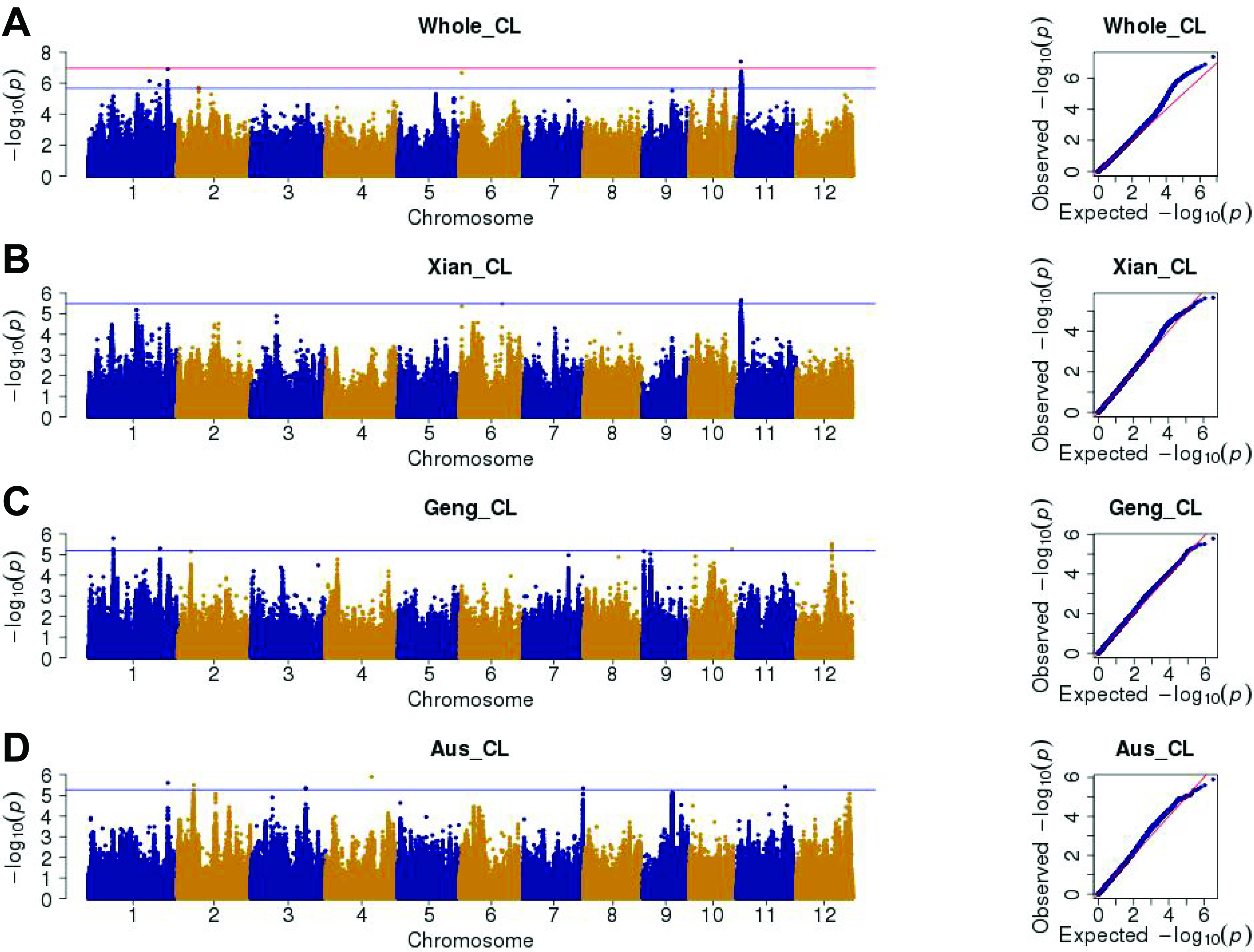

Supplement: Supplementary file 10 — Additional file 10 : Figure S1. Manhattan and quantile-quantile plots for culm length based on the whole, Xian, Geng, and Aus panels using EMMAX. [file 12284_2019_351_MOESM10_ESM.tif]

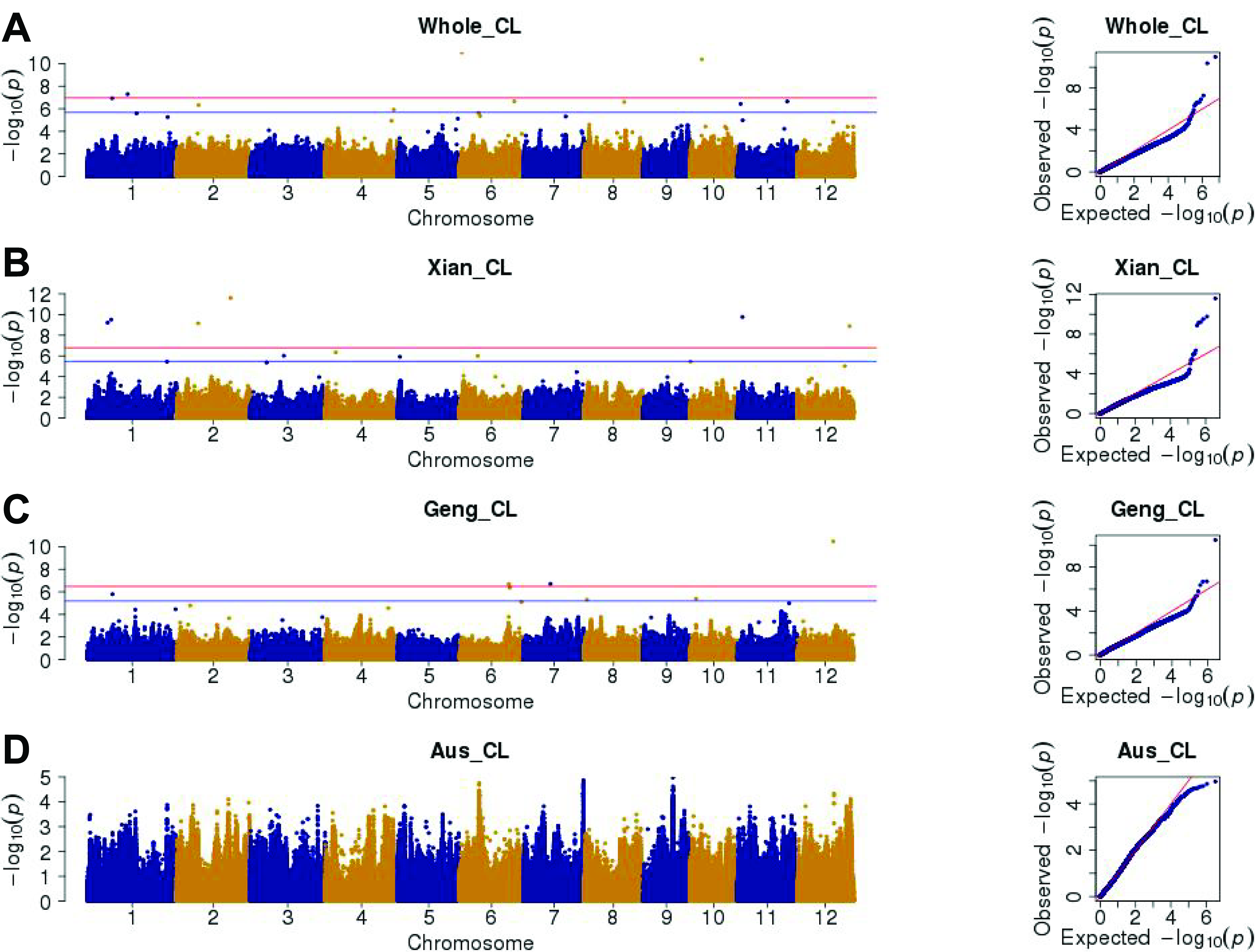

Supplement: Supplementary file 11 — Additional file 11 : Figure S2. Manhattan and quantile-quantile plots for culm length based on the whole, Xian, Geng, and Aus panels using FarmCPU. [file 12284_2019_351_MOESM11_ESM.tif]

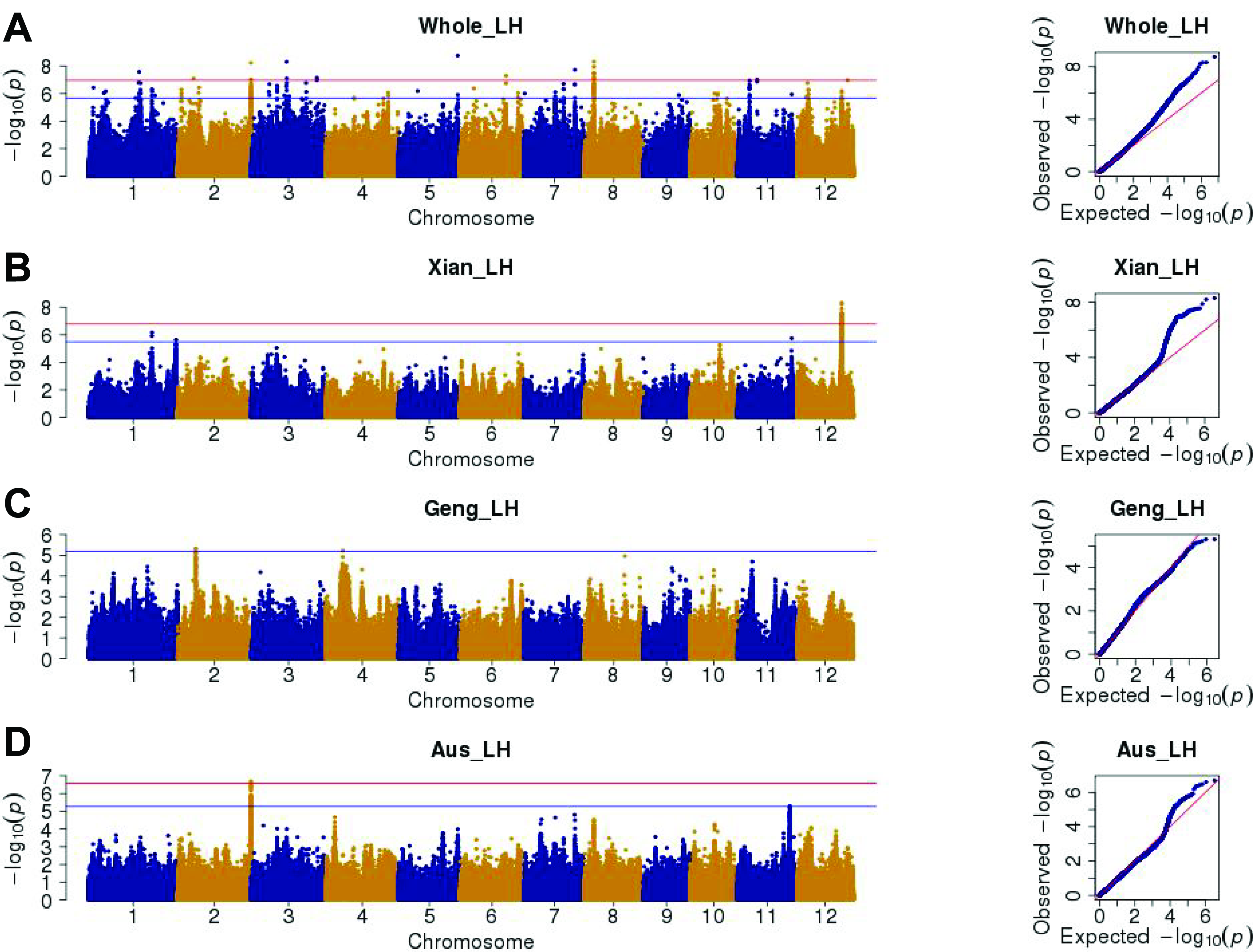

Supplement: Supplementary file 12 — Additional file 12 : Figure S3. Manhattan and quantile-quantile plots for lesion height based on the whole, Xian, Geng, and Aus panels using EMMAX. [file 12284_2019_351_MOESM12_ESM.tif]

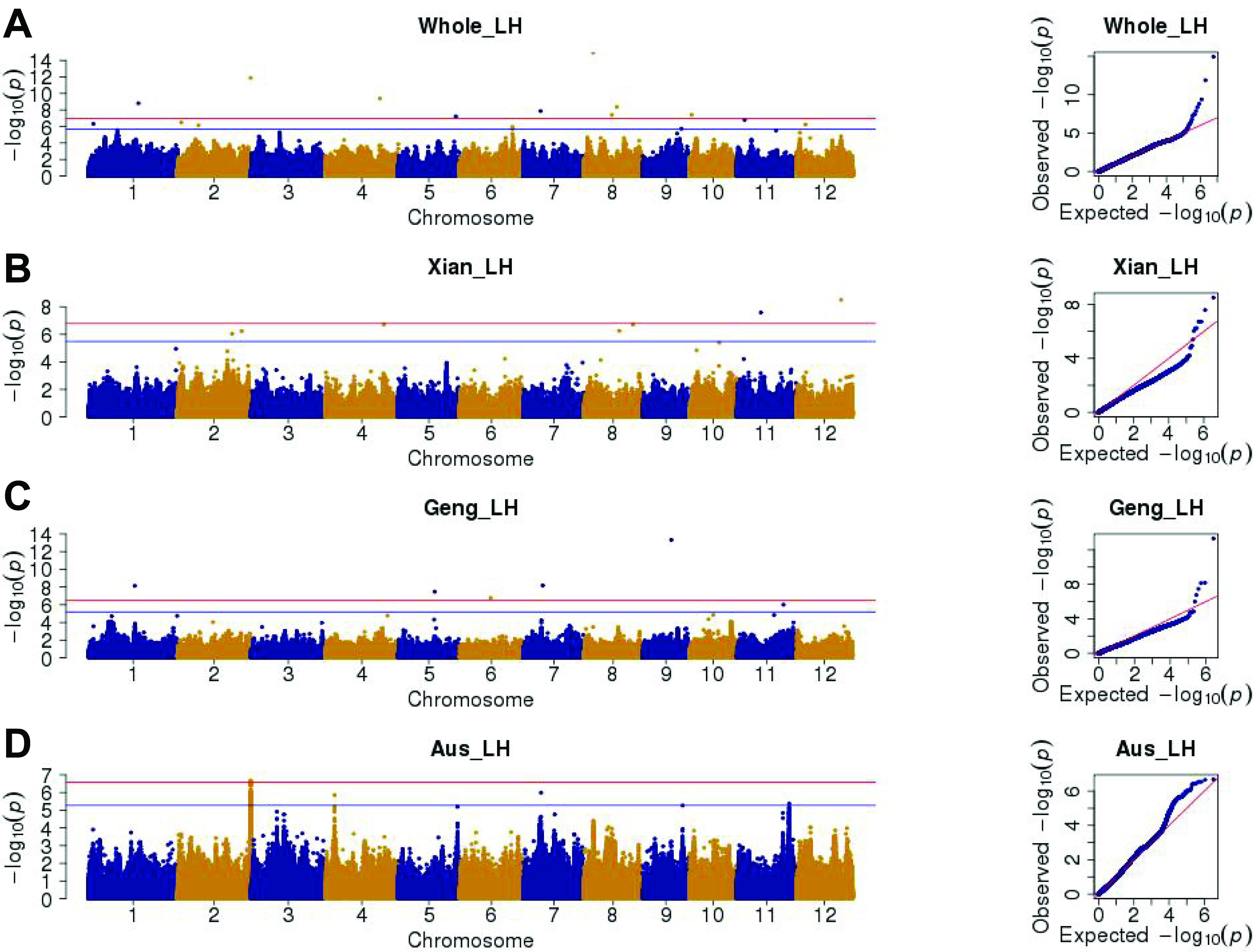

Supplement: Supplementary file 13 — Additional file 13 : Figure S4. Manhattan and quantile-quantile plots for lesion height based on the whole, Xian, Geng, and Aus panels using FarmCPU. [file 12284_2019_351_MOESM13_ESM.tif]

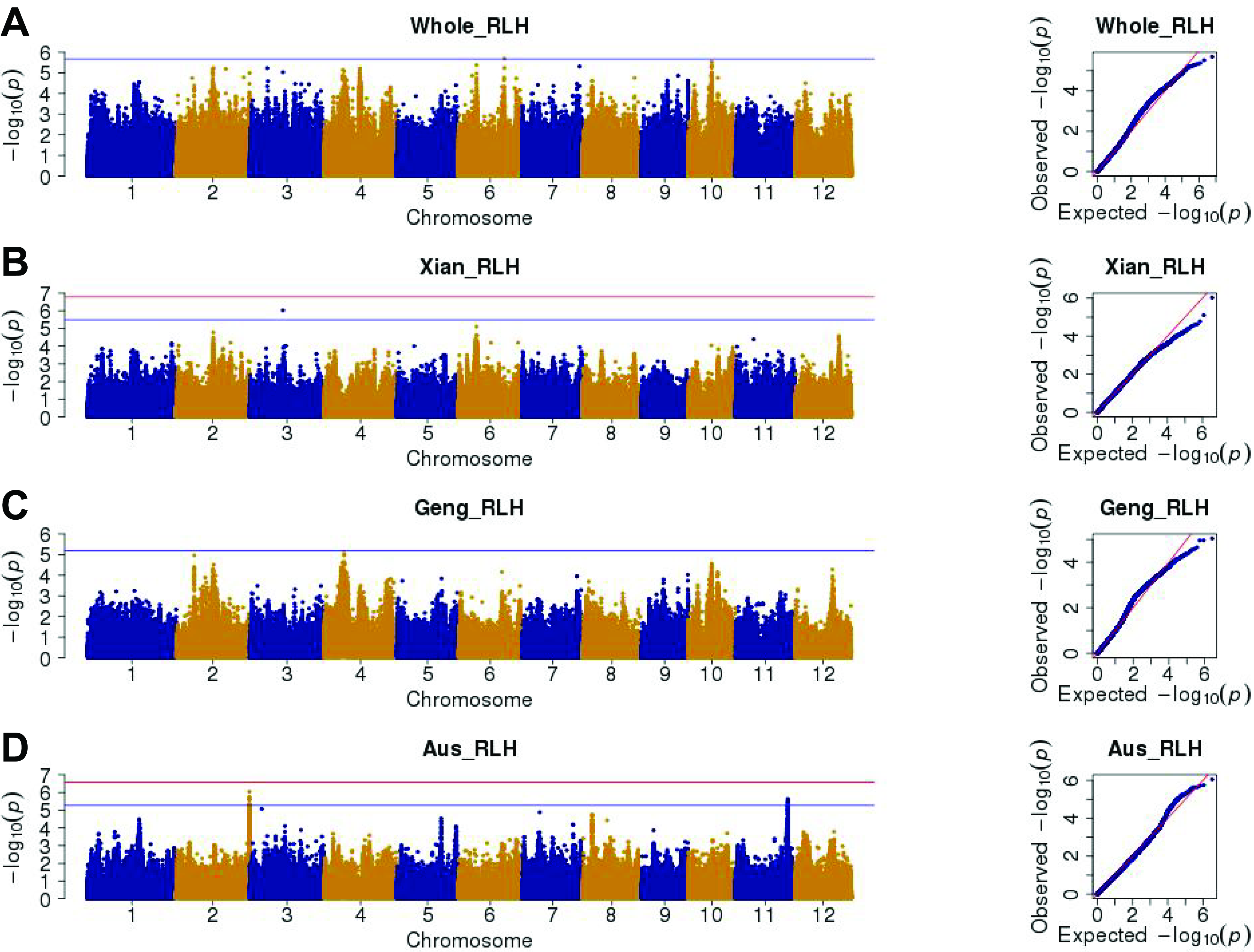

Supplement: Supplementary file 14 — Additional file 14 : Figure S5. Manhattan and quantile-quantile plots for relative lesion height based on the whole, Xian, Geng, and Aus panels using EMMAX. [file 12284_2019_351_MOESM14_ESM.tif]
